# Aligning public health and public safety: Probation as a touchpoint to identify and link patients with opioid use disorder to opioid agonist treatment

**DOI:** 10.1371/journal.pgph.0002349

**Published:** 2023-11-01

**Authors:** Daniel J. Bromberg, Samy J. Galvez de Leon, Taylor Litz, Lyu Azbel, Amanda R. Liberman, Maxim Polonsky, Sergii Dvoriak, Nataliia Saichuk, Faye Taxman, Frederick L. Altice

**Affiliations:** 1 Yale University School of Public Health, New Haven, Connecticut, United States of America; 2 Yale Center for Interdisciplinary Research on AIDS, Yale University, New Haven, Connecticut, United States of America; 3 Yale University School of Medicine, New Haven, Connecticut, United States of America; 4 Ukrainian Institute on Public Health Policy, Kyiv, Ukraine; 5 George Mason University Schar School of Policy and Government, Arlington, Virginia, United States of America; University of California San Diego, UNITED STATES

## Abstract

**Background:**

People in criminal justice settings (CJS) have high rates of opioid use disorder (OUD) and HIV. Probation is part of the CJS and congregates many individuals with high rates of mental health and substance use disorders relative to the general population; nevertheless, probation remains a major improvement to incarceration. As a steppingstone to full decarceration efforts, community supervision settings like probation can be leveraged as “touchpoints” to identify and link people with OUD (and other co-morbid conditions) to treatment and reduce criminal activity.

**Methodology:**

To determine the feasibility of a modified screening, brief intervention and referral to treatment (SBIRT) strategy to link probationers to opioid agonist therapies (OAT) in the newly created probation system in Ukraine, we conducted a single-arm SBIRT intervention in eight probation centers in four Ukrainian administrative regions. For those screening positive for OUD, interest in OAT was assessed before and after a brief intervention. Those interested in OAT were referred to community OAT services. Participants with OUD also underwent HIV testing.

**Principal findings:**

Of the 1,298 consecutive individuals screened, 208 (16.0%) met criteria for opioid dependence. Of these, 122 (58.7%) enrolled in brief intervention, of which 54 (44.3%) had HIV and 14 (25.9%) of these were newly diagnosed. After the brief intervention, interest in starting OAT increased significantly from a median of 7.0 to 8.0 (P = <0.001) using a 10-point scale. Thirty (N = 30; 24.6%) of the enrolled participants initiated OAT and 21 of these (70%) were retained in treatment for 6 months.

**Significance:**

The prevalence of OUD (and HIV) is high among people in probation in Ukraine. SBIRT can identify a large number of people eligible for OAT, many of whom were willing to initiate and remain on OAT. Integrating SBIRT into probation can potentially assist with OAT scale-up and help address HIV prevention efforts.

## Introduction

MOUD, especially opioid agonist treatments (OAT) like methadone and buprenorphine, have the benefit of reducing drug use, crime and incarceration (public safety), but also reduce mortality and transmission of HIV and HCV, while improving health-related quality of life, employment and social functioning (public health) [1]. Most efforts that involve implementing MOUD in CJS have focused on carceral settings like prisons and pre-trial detention centers (i.e., jails) [2]. The CJS extends to community settings like probation, a potential touchpoint for identifying and improving access to treatment for people with opioid use disorder (OUD). Prisons and jails have decreased their censuses substantially to reduce incarceration levels (i.e., decarcerate). Many of the people who would otherwise have been incarcerated have been redirected to probation. The trans-institutionalization of CJS-involved persons from carceral to probation settings has resulted in large numbers of people with or at risk for several health conditions needing public health attention like OUD, HIV, HCV and tuberculosis concentrated in probation [3, 4]. Yet probation, unlike prisons and jails where provision of health and prevention services are recommended, only supports public safety efforts.

A scoping review of interventions for drug use among probationers found only two studies, both from the U.S., that focused on MOUD among people supervised in probation. These studies, however, were limited by identifying only those that sought treatment, and did not include opportunities to identify individuals who may benefit from treatment nor did it involve referrals to treatment [5, 6]. Aligning public safety and public health by targeting probation settings for active screening will have the most benefit where diseases OUD, HIV, HCV, tuberculosis and incarceration are highly prevalent and syndemic.

The Eastern European and Central Asian (EECA) region has among the highest prevalence globally [1] of opioid use disorder (OUD), HIV, HCV, tuberculosis and incarceration [7–9]. OAT is an evidence-based strategy for controlling HIV [10], HCV [11, 12], and tuberculosis [13] and, when adequately scaled, substantially reduces HIV incidence and mortality [7]. Moreover, OAT remains the most effective treatment for OUD for criminal justice-involved persons [14], and is associated with reductions in criminal activity and reincarceration [1]. OAT coverage in EECA, however, remains well below internationally recommended targets [15, 16] and is often available only as pilot programs or is not present at all [7].

Ukraine is a LMIC with the highest HIV prevalence (1.2%) among adults in Europe, concentrated among PWID, with biobehavioral surveys (conducted via respondent-driven sampling, therefore collecting data on “hidden” populations) showing increasing HIV incidence and mortality due to low levels of identification of PWH and treatment of OUD [17]. Due to the criminalization of drug use (opioids are almost entirely injected in Ukraine), PWID in Ukraine have historically been concentrated in prisons, thereby also concentrating people with syndemic conditions like HIV and HCV [18, 19]. OAT scale-up has been hindered by negative attitudes toward and misinformation about it among PWID [20–23] generally and, scale-up of OAT in prisons has been hindered by criminal subculture that affects living conditions for those that start OAT [20, 24–29] and strongly negative attitudes toward, and myths about, OAT.

In 2015, Ukraine’s incarceration rate was among the 10 highest globally [7]. To better align itself with Western European practices, a new probation program in Ukraine was started in August 2015. Incarceration subsequently decreased from 324 to 195 per 100,000 population [7], while probation increased its census by 57,396 to support public safety efforts. Before probation, 48.7% of incarcerated people were people who inject drugs (PWID) and 19.4% had HIV [30]. As probation in Ukraine grew, trans-institutionalization of large numbers of persons with OUD at high risk for HIV and HCV were no longer interfacing with carceral settings that provide a minimum standard for health and prevention services [31, 32]. Such guidance for probation settings, does not exist as people are perceived to be able to freely access community prevention and treatment services [33].

Though probation may reduce the harm from residing in and being released from carceral settings, it does not represent full freedom from the carceral system (decarceration). In addition to people on probation having higher prevalence of substance use and psychiatric disorders relative to the general population [34], the experience of probation can, for example in the U.S., exacerbate inequality as it is disproportionately levied on racial and ethnic minorities [35–37]. Until further progress with decarceration efforts, probation is an intermediate step toward reducing the negative consequences of the CJS, but it may reduce harms further by aligning public safety and public health goals if it can improve access to evidence-based treatments.

In Ukraine where OAT coverage nationally is low (<5%) and nearly half of people with HIV (PWH) remain undiagnosed, the probation system is ideally situated to identify and link highly vulnerable people to OAT and HIV care, as long as services are accessible. As PWID seldom rely on care in traditional primary care settings, “touchpoints” like syringe services programs (SSP), emergency departments, and prisons and jails are uniquely positioned for engaging PWID [38], yet strategies to involve probation within the CJS are either non-existent or under-utilized. We implemented a screening, brief intervention and referral to treatment (SBIRT) strategy in the newly formed probation system in the low-middle income country (LMIC) of Ukraine with alarmingly high rates of OUD and HIV.

To test the hypothesis that probation is an opportune setting, or “touchpoint”, to identify and treat people with OUD, SBIRT was introduced into probation offices in several cities throughout Ukraine. SBIRT is an evidence-based approach designed to identify patients with OUD, inform and motivate them to start OAT, and link them to care. Although SBIRT is generally effective at improving uptake of alcohol-related services [39], its benefits for other substance use disorders have been called into question [40]. The goal of this study, therefore, was to assess the effectiveness of a modified SBIRT strategy that involves a “green-light” referral to OAT (similar to clinical practice guidelines) for anyone identified with OUD [21].

## Methods

### Study setting

This study was conducted at eight probation centers in four Ukrainian administrative regions between January 2017 to February 2018: Kyiv city (N = 3), Mykolaiv oblast (N = 2), Dnipro oblast (N = 2), and Sumy oblast (N = 1).

### Participants

Inclusion criteria included being under probation supervision and: 1) 18 years or older; 2) live within 30 kilometers of an OAT site; and 3) not have received OAT for at least 15 days. Individuals screening positive for OUD were further enrolled for brief intervention and linkage to treatment. Patients who did not screen positive for OUD were not included in further aspects of the study (i.e. the brief intervention).

### Procedures

Trained research assistants screened consecutive entrants to the probation site using a modified, single-item screening question (SISQ) [41] that has been modified for OUD and is 86% sensitive [42], and oversaw informed consent procedures. Anyone screening positive then underwent confirmation for opioid dependence using the brief Rapid Opioid Dependence Screen [43], and were asked to return at their next probation visit (7–28 days) for enrollment and baseline survey completion, which assessed baseline characteristics and interest in OAT. All interviews were self-administered using REDCap on tablet computers. After completing the baseline survey, the research assistant delivered a 10-minute brief intervention using motivational interviewing techniques. The intervention involved informing participants on the risks of opioid use by illustrating the potential hazards and adverse health consequences and the relative benefits of OAT (e.g., prevention of overdose, HIV risk reduction and criminal activity) relative to continued injection of opioids. OAT in Ukraine, aside from some newly emerging private clinics [44, 45], is provided free by the Ministry of Health. The brief intervention was audio-recorded for quality control and fidelity assessment, with coaching provided to intervention deliverers. After the brief intervention, participants were reassessed for their interest in OAT. Individuals could be linked to OAT either before or after the brief intervention, depending on their level of interest.

Approximately 30 days after the initial brief intervention (median 21 days), participants completed a second interview and brief intervention. For those already on OAT, the brief intervention focused on retention. Patients were followed for 6 months to examine linkage to and retention in OAT.

### Laboratory procedures

All participants with OUD and consented for the brief intervention underwent combination rapid testing for HIV, HBV, HCV, and syphilis at baseline and after 6 months. All participants received post-test counseling and referral to medical and treatment services. In addition, participants underwent urine drug testing for opioids at baseline and at months 1, 3, and 6.

### Measures

Initiation of OAT was examined within 6 months of the baseline interview. Objective initiation dates were verified using Ukraine’s national database for monitoring OAT (i.e, SyrEx) which we have used previously [46]. As part of the brief intervention, interest in, difficulty in accessing, and importance of initiating OAT were measured before and after the intervention. Interest in OAT was assessed using a scale ranging from 0 indicating no interest and 10 indicating the greatest possible level of interest. Interest in OAT was defined as a score of ≥1 on the 10-point scale.

Additional standardized measures included a standardized 8-item, 5-point Likert scale assessing OAT attitudes and knowledge with higher numbers indicating greater knowledge and positive attitudes. OAT attitudes (*α* = 0.74) included two items that measured participant’s favorable attitudes towards OAT. OAT knowledge (*α* = 0.93) used 6 items related to participant’s knowledge about the positive effects of OAT on health and criminal activity outcomes. Moderate to severe depression was assessed if the score on the 10-item Center for Epidemiological Studies-Depression (CES-D) scale was ≥10 [47].

### Statistical analysis

Data analyses were conducted using SAS 9.4. Categorial variables were compared by using χ2 or Fisher’s exact test, as appropriate. Continuous variables were compared by using the t-test or Mann Whitney U test, as appropriate. Normality was tested with the Shapiro-Wilk test. Differences in OAT interest scores before and after brief intervention were tabulated, and statistical significance was determined using a Wilcoxon signed-rank test. Statistical significance was defined as p<0.05.

### Ethics statement

The study is registered at www.clinicaltrials.gov (NCT 04947475) and was approved by institutional review boards at Yale University (1407014374) and the Ukrainian Institute on Public Health Policy.

## Results

### Disposition of participants

**Fig 1:**
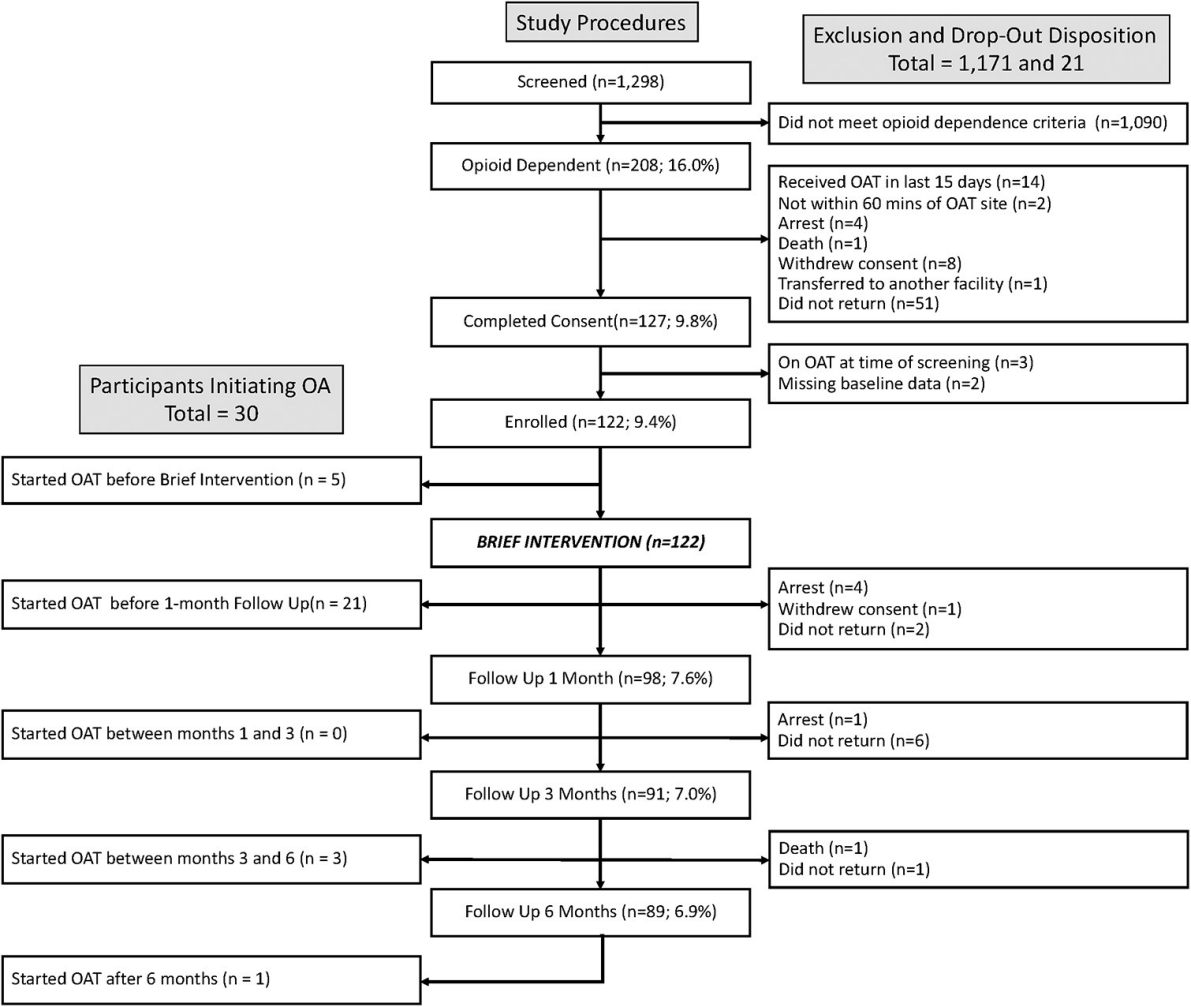
Participant disposition.

Of the 1,298 consecutive individuals screened in probation 16.0% (N = 208) met screening criteria for OUD and 193 met diagnostic criteria for OAT; 122 of (63.2%) eligible participants enrolled in the brief intervention and linkage component. Participant characteristics are presented in Table 1. Disposition of participants of screening, eligibility, and study event completion are presented in Fig 1. No significant differences in age or sex were noted between participants and eligible non-participants. Of those enrolled, 121 (99.2%) completed the brief intervention and 103 (84.4%) completed the more intensive second intervention.

**Table 1:** Participant characteristics, stratified by Initiation of OAT (N = 122).

Characteristic		Started OAT		*p*-value
	Total N = 122	Yes N = 30	No N = 92	
	N (%)	N (%)	N (%)	
*City*				< .001
Kyiv	48 (39.3)	16 (53.3)	32 (34.8)	
Dnipro	43 (35.3)	1 (3.3)	42 (45.7)	
Mykolaiv	26 (21.3)	13 (43.3)	13 (14.3)	
Sumy	5 (4.1)	0	5 (5.4)	
*Sex*				1.00
Male	105 (86.1)	26 (86.7)	79 (85.9)	
Female	17 (13.9)	4 (13.3)	13 (14.1)	
*Age*	37.0 ± 7.2	36.5 ± 5.5	37.1 ± 7.7	0.64
*Marital Status*				0.20
Partnered	53 (43.4)	10 (33.3)	43 (46.7)	
Not partnered	69 (56.6)	20 (66.7)	49 (53.3)	
*Children*				0.76
Yes	58 (47.5)	15 (50.0)	43 (46.7)	
No	64 (52.5)	15 (50.0)	49 (53.3)	
*Education*				0.63
Less than high school	24 (19.7)	5 (16.7)	19 (20.7)	
Completed high school or higher	98 (80.3)	25 (83.3)	73 (79.4)	
*Housing*				0.41
Own residence (rent/own)	25 (20.5)	7 (23.2)	18 (19.6)	
Friend or Relative’s home	92 (75.4)	23 (76.7)	69 (75.0)	
Unstable	5 (4.1)	0 (0.0)	5 (5.4)	
*Employment*				0.25
Full time/some employment	68 (55.7)	14 (46.7)	54 (58.7)	
Unemployed	54 (44.3)	16 (53.3)	38 (41.3)	
*Previously Incarcerated*				0.06
Yes	77 (63.1)	15 (50.0)	62 (68.9)	
No	43 (35.2)	15 (50.0)	28 (31.1)	
*Registered at Narcology addiction treatment center*				0.25
Yes	72 (59.0)	15 (50.0)	35 (38.0)	
No	50 (41.0)	15 (50.0)	57 (62.0)	
*Previously on OAT*				0.06
Yes	13 (10.7)	6 (20.0)	7 (7.6)	
No	109 (89.3)	24 (80.0)	85 (92.4)	
Self-rated health				0.34
Poor/Fair	85 (69.7)	23 (76.7)	62 (67.4)	
Greater than fair	37 (30.3)	7 (23.3)	30 (32.6)	
**Medical History**				
*HIV antibody*				0.33
Positive	54 (44.3)	11 (36.7)	43 (46.7)	
Negative	68 (55.7)	19 (63.3)	49 (52.3)	
*Hepatitis B antibody*				0.25
Positive	1 (0.82)	1 (3.3)	0 (0.0)	
Negative	121 (99.2)	29 (96.7)	92 (100.0)	
*Hepatitis C antibody*				0.20
Positive	82 (67.2)	23 (76.7)	59 (64.1)	
Negative	40 (32.8)	7 (23.3)	33 (35.9)	
*Syphilis antibody*				0.60
Positive	5 (4.1)	2 (6.7)	3 (3.3)	
Negative	117 (95.9)	28 (93.3)	89 (96.7)	
Depression*, *moderate to severe*				0.07
Yes	72 (59.0)	22 (73.3)	50 (54.4)	
No	50 (41.0)	8 (26.7)	42 (45.7)	
**Drug Use**				
*Overdose (last 6 months)*				0.04
Yes	12 (9.84)	0	12 (13.0)	
No	110 (90.2)	20 (100)	80 (87.0)	
*Injection frequency (last 30 days)*				0.29
Daily	55 (45.1)	11 (36.7)	44 (47.8)	
Less than daily	67 (54.9)	19 (63.3)	48 (52.2)	
*Drug Abuse Severity*				0.15
Low/Moderate	4 (3.3)	0	4 (4.4)	
Substantial	59 (48.4)	19 (63.3)	40 (43.5)	
Severe	59 (48.4)	11 (36.7)	48 (52.2)	
**Social Support**				
*Social support scale score*	3.49 (1.1)	3.37 (1.3)	3.5 (1.0)	0.57
*Receive social support from probation officers*				0.43
Yes	24 (19.7)	4 (13.3)	20 (21.7)	
No	98 (80.3)	26 (86.7)	72 (78.3)	
*Want support from probation officer for OAT*				0.03
Yes	41 (33.6)	15 (50.0)	26 (28.3)	
No	81 (66.4)	15 (50.0)	66 (71.7)	

Note: Data are presented as mean (SD) for continuous variables or number and percentage for categorical variables

* Cut-off criteria ≥10

### Participant characteristics

The mean age of participants was 37.0 years (range 20–54 years) and 86.1% were male. Overall, two-thirds of participants were antibody positive for HCV, more than half met screening criteria for moderate to severe depression, and 44% were positive for HIV. There was no significant difference between those who started OAT and those who did not start OAT in demographic and medical history characteristics. Participants who experienced an overdose in the previous 6 months were significantly more likely to start OAT compared to those who did not (0% vs 13.0%, p = 0.04). Fifty-four (44.3%) of the participants tested positive for HIV, with 14 (25.9%) of them being newly diagnosed during SBIRT procedures (See Table 2).

**Table 2:** HIV status self-report by HIV antibody test result.

		HIV test result		Total
		Positive	Negative	
HIV status self-report	Positive	40 (74.1)	0 (0.0)	40
	Negative/Don’t know	14 (25.9)	68 (100.0)	82
Total		54	68	122

### Treatment outcomes

**Fig 2:**
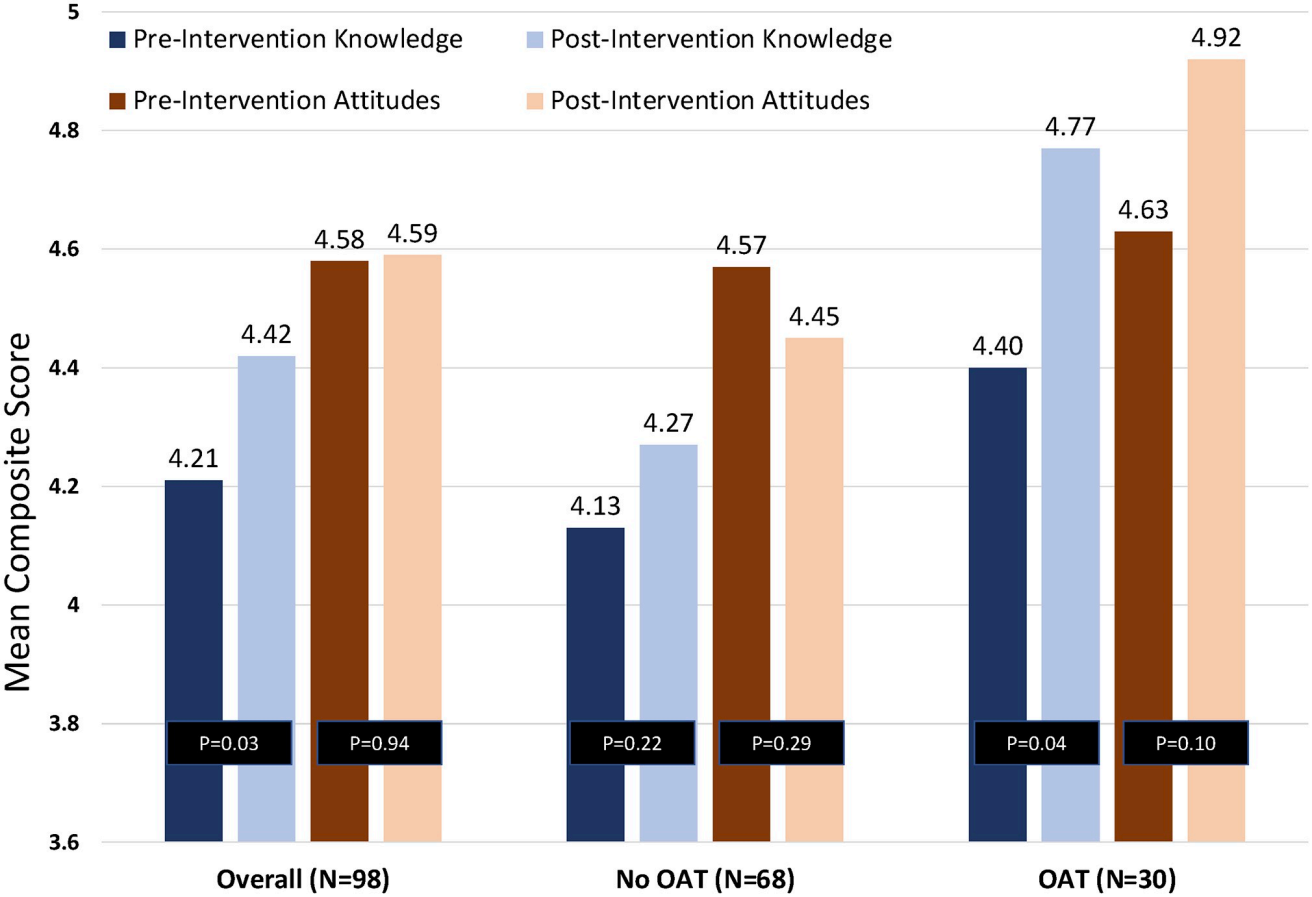
Changes in knowledge and attitudes scores from baseline to month 1, stratified by OAT initiation. OAT = Opioid Assisted Therapy.

**Fig 3:**
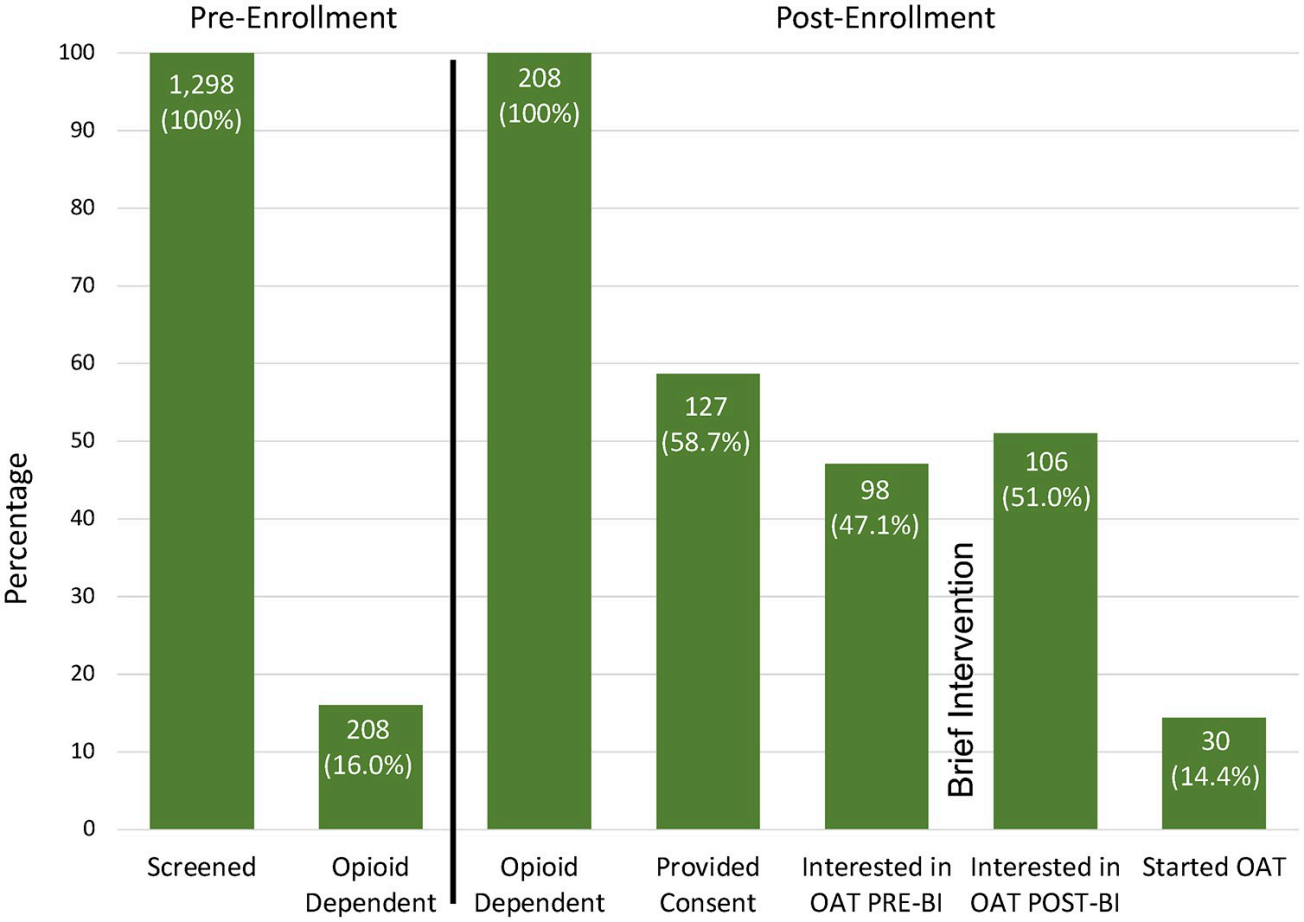
Opium continuum of care cascade for people experiencing probation in Ukraine. OAT = Opioid Agonist Therapy, BI = Brief Intervention.

**Fig 4:**
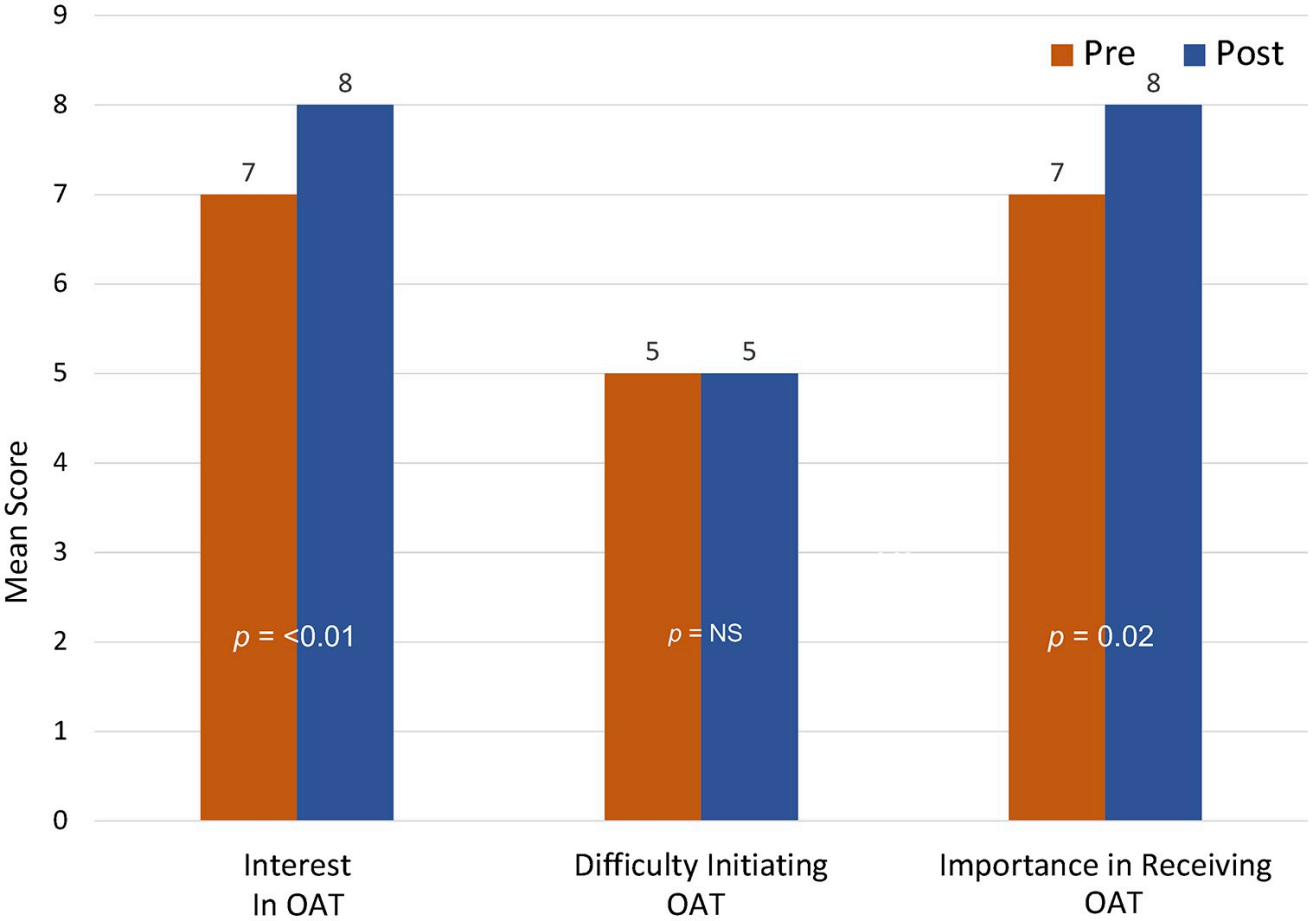
OAT interest, difficulty, and importance scores before and after brief intervention (N = 122).

During the initial assessment, 86.7% (N = 98) met interest criteria for initiating OAT and were offered immediate access to treatment. Upon completion of the brief intervention, the proportion of those interested in OAT initiation increased to 93.0% (N = 106) and the median interest score increased from 7.0 to 8.0 (p<0.001) on the 10-point scale. Similarly, the median score for importance in receiving OAT increased from 7.0 to 8.0 (P = 0.02) (See Table 3 and Fig 2). Participants’ attitudes towards treatment remained mostly unchanged after the brief intervention, except for a stronger preference for methadone over buprenorphine (p<0.001), and a more cemented belief that OAT may aid in preventing HIV (p = 0.05) (See Tables 4 and 5, Figs 3 and 4). Reasons participants initiated OAT are presented in Table 6, and reasons for not being interested in OAT are presented in Table 7. Correlates of OAT initiation included post-intervention importance of OAT, as well as previous OAT entry attempts (Table 8).

**Table 3:** Changes in interest in, difficulty in accessing, and importance to initiate opioid agonist therapies before and after a brief intervention (N = 113).

	Pre-Intervention	Post-Intervention	*p*-value
Interest Score, median (IQR)	7.0 (4–10)	8.0 (5–10)	**< .001**
Difficulty Score, median (IQR)	5.0 (2–7)	5.0 (1–7)	0.90
Importance Score, median (IQR)	7.0 (3–10)	8.0 (3–10)	**0.02**

**Table 4:** Mean OAT attitude and knowledge scores at baseline vs. month 1 follow up (n = 97).

Statement^a^	Baseline Mean (SD)	Follow Up Mean (SD)	*p*-value	Effect size Cohen’s *d*
OST services should be available in the community so that all people who suffer from opioid addiction and OST can receive it.	4.66 (0.85)	4.71 (0.72)	0.59	
OST services should be introduced into prisons so that all inmates who suffer from opioid addiction and want opioid substitution therapy can receive it.	4.49 (1.11)	4.47 (1.18)	0.86	
OST reduces opioid dependent individuals’ risk of acquiring or transmitting HIV.	4.38 (1.17)	4.58 (0.93)	**0.05**	**-0.20**
OST reduces addicts’ criminal activities.	4.30 (1.20)	4.52 (0.93)	0.06	
OST improves adherence to HIV medications in HIV-infected opioid dependent individuals.	4.21 (1.17)	4.42 (0.99)	0.08	
OST reduces opioid dependent individuals’ consumption of illicit opiates.	4.18 (1.29)	4.38 (1.07)	0.09	
OST increases opioid dependent patients’ adherence to tuberculosis medication	4.09 (1.27)	4.31 (1.07)	0.11	
OST decreases opioid dependent individuals’ risk of dying from overdose.	4.13 (1.36)	4.32 (1.18)	0.13	
For people who have opioid addiction, it would be much better to treat them with buprenorphine rather than methadone.	2.93 (1.52)	2.28 (1.46)	**< .001**	**0.36**

^a^ Range of responses is 1–5 (1 = strongly disagree, 5 = strongly agree)

**Table 5:** Perceived treatment effectiveness (How effective are each of these approaches in treating opioid dependence?).

Approaches*		Started OAT		p-value
	Total	Yes	No	
	N = 122	N = 30	N = 92	
	Mean (SD)	Mean (SD)	Mean (SD)	
Opioid Substitution Therapy	3.93 (1.07)	4.37 (0.67)	3.78 (1.14)	**< .01**
Family Support	3.33 (1.28)	3.30 (1.47)	3.35 (1.23)	0.86
New Environment	3.21 (1.37)	3.03 (1.56)	3.27 (1.30)	0.41
Religion	2.75 (1.35)	2.87 (1.28)	2.71 (1.38)	0.60
Detox	2.37 (1.31)	2.37 (1.27)	2.37 (1.33)	0.99
Rehabilitation	2.48 (1.42)	2.50 (1.46)	2.47 (1.42)	0.94
Incarceration	2.43 (1.42)	1.80 (1.13)	2.64 (1.46)	**< .01**
Employment	2.34 (1.33)	2.53 (1.43)	2.27 (1.29)	0.35
Treatment with medication (other than OST)	2.09 (1.26)	2.20 (1.27)	2.05 (1.26)	0.58
Alternative Medicine	1.24 (0.69)	1.20 (0.48)	1.39 (0.74)	0.19

*Range of responses 1–5 (1 = Not effective, 5 Very effective)

**Table 6:** Reasons for initiating OAT among people who inject drugs who received OAT in the past (n = 13).

Reasons	N (%)
Financing drug consumption was too expensive	10 (76.9)
I was using too much and wanted to reduce my drug use	9 (69.2)
Wanted to make a change in the circles I was moving in	8 (61.5)
Concerned of being arrested/imprisoned	8 (61.5)
Wanted to improve my health	6 (46.2)
I wanted to stop using illegal opioids permanently	6 (46.2)
Wanted to stop committing crimes for my habit	6 (46.2)
Wanted to take better care of my family	3 (23.1)
I wanted to be able to work again	3 (23.1)
I needed a break from my habit because life was too chaotic	2 (15.4)
Afraid of losing my job	2 (15.4)
I wanted to get clean to get my HIV treated	2 (15.4)
Afraid of getting an infection or contracting a disease	2 (15.4)
I was afraid I might overdose	2 (15.4)
My family wanted me to start	1 (7.7)

**Table 7:** Reasons for not being interested in OAT among people who inject drugs who have never been on OAT (N = 34).

Reasons	N (%)
I think methadone is just another drug	26 (76.5)
I will have to take methadone all my life	15 (44.1)
My friends had a bad experience on OAT	15 (44.1)
Methadone will destroy my health	11 (32.4)
Did not want to get registered	9 (26.5)
Do not like the rules of the program (hours/location)	7 (20.6)
My family and friends have negative feelings about OAT	4 (11.8)
Methadone is free and therefore bad quality	3 (8.8)
I don’t want my friends to know I’m on treatment	3 (8.8)

**Table 8:** Correlates of initiating OAT among people who inject drugs (N = 122).

Variable	*OR (95% CI)*	*aOR (95% CI)*
Importance (Post-intervention)	1.462 (1.183, 1.806)	1.401 (1.114, 1.762)*
Previous OAT entry attempts	5.769 (2.360, 14.100)	3.959 (1.339, 11.711)*
Severe Drug Abuse	0.531 (0.227, 1.239)	-
Previously Incarcerated	0.452 (0.194, 1.050)	-
Depression	2.310 (0.932, 5,723)	-

*p < .01

OAT Initiation:

• Of the 30 participants who started OAT during the study, 9 (30%) stopped OAT before their month 6 follow up interview.

• The remaining 21 participants were still on OAT at the time of their month 6 follow up.

Overall, 30 (24.6%) individuals started OAT. Five started immediately after screening and before the brief intervention, 21 started within 30 days after the brief intervention and 4 started after 30 days. Results did not differ by HIV status. Among participants who initiated OAT, 80% were participants who had never received OAT previously. In the multivariable analysis, having previously received OAT portended a 4-fold likelihood of initiating OAT, while increasing levels of importance for OAT portended a 40% increased likelihood of initiating OAT. Of the 30 that initiated OAT, 21 (70%) remained on treatment for 6 months (See Fig 3).

## Discussion

Until decarceration efforts are fully supported, probation represents an opportunity to align public safety and public health goals and can serve as a “touchpoint” to engage PWID in prevention and treatment services. As LMIC like Ukraine begin the process toward decarceration through the introduction of interim programs, probation can be leveraged to identify high-risk individuals with OUD (and HIV) and link them to needed treatment.

Integrating screening for OUD along with HIV and HCV provides an additional opportunity to address individual and public health priorities, especially if participants can be linked to appropriate treatment and prevention services (e.g., antiretroviral therapy and pre-exposure prophylaxis for HIV, respectively, and direct-acting antiviral medications for HCV). Importantly, the prevalence of OUD in Ukrainian probation settings is 16%, 16-fold higher than in the community [7]. Also, the brief intervention significantly increased interest in starting OAT, especially by improving accurate knowledge, but not attitudes, about OAT. Moreover, the improved knowledge about OAT translated to initiation of an evidenced-based treatment for OUD for many, especially in a setting with negative attitudes toward OAT.

This SBIRT strategy that focused on practice guidelines linked 25% of study participants to OAT, the most effective [48] and cost-effective [49] strategy to control HIV for Ukraine. Moreover, retention on OAT was high (70%) over 6 months of observation. If hypothetically our sample were representative of the overall probation population in Ukraine, adoption of this SBIRT strategy by probation officers and implemented throughout Ukraine the ~60,000 people under community supervision, would translate to ~9,600 with criteria for opioid dependence and as many as 2,342 could be initiated on OAT. These numbers do not incorporate the dynamic of new people entering the probation system. Further research with a randomly selected, representative sample can elucidate the exact extent to which SBIRT might scale up OAT in Ukraine and elsewhere. Even to the extent that it would be less effective, SBIRT may likely still be very cost-effective as it is not expensive to implement.

These findings diverge from a 2015 systematic review and meta-analysis [39, 50] that found no evidence that brief interventions significantly increase treatment uptake, though patients with OUD did not figure prominently in these studies, and none of them included probation settings [51]. Two very important issues may have contributed to the improved outcomes here. First, this justice-involved population may have opted for OAT to reduce criminal activity and to avoid incarceration. Second, the study focused only on patients with OUD, as there is an evidenced-based treatment for this condition. Third, there was no comparison group to determine the extent to which individuals on probation may have sought treatment independent of the brief intervention as five participants opted for treatment without further intervention. The extent to which screening alone contributed to treatment initiation is not known. This last observation is supported in that the brief intervention did not substantially improve attitudes towards OAT, aside from an increased preference for methadone over buprenorphine and increased the belief that OAT helps prevent HIV transmission.

Of remaining concern is that most PWID in Ukraine from national surveys would not initiate OAT [29], mostly due to misinformation and myths being major barriers to OAT uptake and retention [21, 22]. This study confirms that these myths and beliefs persist, mostly unchanged despite the intervention, and may require different interventions in order to produce a significant change in beliefs. This is especially true as three-quarters of participants did not start OAT, despite its documented benefits. An in-depth examination of reasons for non-uptake post intervention should be conducted. Previous qualitative studies have discovered the complex social dynamics underlying non-uptake of methadone in prisons [7, 24, 30, 52–55], yet the social dynamics of probation are unknown.

In addition to suboptimal OAT coverage, only 67% of the estimated 250,000 PWH are diagnosed in Ukraine. In probation in Ukraine, not only were one third (32.8%) of people with OUD found to have HIV, a similar proportion found in prison settings [30], but over a quarter (25.9%) were undiagnosed, making probation sentinel surveillance sites for co-screening for HIV. Though this study only secondarily screened for HIV if probationers met criteria for OUD, the findings here confirm that SBIRT benefits are optimized when syndemic conditions are bundled during screening [56] and future efforts might include screening for a number of syndemic conditions associated with criminal justice (e.g., OUD, HIV, HCV, depression, and other needs) [57].

Despite the many important findings, there are limitations. Of the 209 who met inclusion criteria, 127 (61.1%) individuals enrolled which limits generalizability. Among the 81 individuals with OUD who did not enroll, 14 individuals were excluded as they had just initiated OAT, thus violating our inclusion/exclusion criteria. The 51 individuals who did not return after screening, however, could have introduced bias as they represented about a quarter (26.6%) of all eligible individuals who underwent screening. These 51 individuals were similar to our final analytic sample in terms of age and sex, but no other variables were available for comparison. Second, though we did not measure it, social desirability bias may have contributed to our findings. For example, several (N = 9) enrolled participants reported never having been on OAT and 6 indicated they were on OAT at baseline, yet the national OAT registry confirmed previous initiation and starting OAT after their screening but before the baseline survey, respectively. Also, many on probation may not have accurately reported their opioid use because of concerns it would affect their probation status. As such, the prevalence of OUD among those on probation may be substantially higher. To what extent social desirability contributed to other self-reports is unclear, but this limitation highlights the need for different types of follow-up procedures in future studies like interviews with peers rather than researchers.

Despite its limitations, this study showed that brief intervention, in the context of a SBIRT strategy is modestly effective at increasing interest in OAT and may increase OAT uptake during probation. Worldwide, there are 2 to 3 times the number of individuals on probation relative to prison, which means that lack of attention to this population is a clear missed opportunity, as it presents a touchpoint for initiation of OAT, and possibly other EBPs. Other types of brief interventions that may target myths and other barriers to OAT uptake include informed decision-making aids [58, 59] which warrant study in this setting.
